# Clinical validation of the HPVIR high-risk HPV test on cervical samples according to the international guidelines for human papillomavirus DNA test requirements for cervical cancer screening

**DOI:** 10.1186/s12985-019-1216-7

**Published:** 2019-08-22

**Authors:** Inger Gustavsson, Riina Aarnio, Mattias Myrnäs, Julia Hedlund-Lindberg, Ongeziwe Taku, Tracy Meiring, Ingrid Wikström, Stefan Enroth, Anna-Lise Williamson, Matts Olovsson, Ulf Gyllensten

**Affiliations:** 1 Department of Immunology, Genetics, and Pathology, Biomedical Center, Science for Life Laboratory Uppsala, Uppsala University, Box 815, SE-75108 Uppsala, Sweden; 2 Department of Women’s and Children’s Health, Uppsala University, SE-751 85 Uppsala, Sweden; 3 Division of Medical Virology, Faculty of Health Sciences, University of Cape Town, Anzio Road, Observatory, Cape Town, 7925 South Africa

**Keywords:** HPV, DNA testing, Primary cervical cancer screening, International guidelines

## Abstract

**Background:**

The indicating FTA card is a dry medium used for collection of cervical samples. HPVIR is a multiplex real-time PCR test that detects 12 high-risk human papillomavirus types (hrHPV) and provides single genotype information for HPV16, − 31, − 35, − 39, − 51, − 56, and − 59 and pooled type information for HPV18/45 and HPV33/52/58. The aim of this study was to evaluate whether a strategy with cervical samples collected on the FTA card and subsequently analysed with the HPVIR test complies with the criteria of the international guidelines for a clinically validated method for cervical screening.

**Methods:**

We performed a non-inferiority test comparing the clinical sensitivity and specificity of the candidate test (FTA card and HPVIR) with a clinically validated reference test (Cobas^®^ HPV test) based on liquid-based cytology (LBC) samples. Two clinical samples (LBC and FTA) were collected from 896 participants in population-based screening. For evaluation of the specificity we used 799 women without ≥ CIN2, and for clinical sensitivity we used 67 women with histologically confirmed ≥ CIN2. The reproducibility was studied by performing inter- and intra-laboratory tests of 558 additional clinical samples.

**Results:**

The clinical sensitivity and specificity for samples collected on the FTA card and analysed using the HPVIR test were non-inferior to samples analysed with the Cobas® HPV test based on LBC samples (non-inferiority test score, *p* = 1.0 × 10^− 2^ and *p* = 1.89 × 10^− 9^, respectively). Adequate agreement of > 87% was seen in both the intra- and inter-laboratory comparisons.

**Conclusions:**

Samples collected on the indicating FTA card and analysed with HPVIR test fulfil the requirements of the international guidelines and can therefore be used in primary cervical cancer screening.

## Background

The use of HPV DNA tests is more effective in primary cervical cancer screening than cytology [1] and national and international guidelines therefore recommend transition to primary screening using HPV test [2, 3]. Over the last decades, Hybrid Capture 2 (HC2, QIAGEN, Germany) and GP5+/6 + −PCR EIA have been the two most widespread HPV tests, and have been clinically validated based on data from randomized control trials [1, 4]. However, a series of additional HPV tests are available but have not been clinically validated [5, 6]. In order to determine if a candidate test fulfil the requirements with regard to clinical sensitivity and specificity for detecting cervical intraepithelial neoplasia grade 2 or worse (≥ CIN2), international guidelines for validation of new tests have been developed [2]. This validation also includes intra-laboratory reproducibility and inter-laboratory agreement. Several other HPV tests, in addition to HC2 and GP5+/6 + −PCR EIA, have been clinically validated according to these guidelines and recommended for use in primary screening [7]. The most common used clinically validated method for HPV testing in screening is based on collection of LBC samples and the Cobas^®^ HPV test [8–10].

HPVIR is a laboratory developed (LDT), multiplex real-time PCR test, detecting and quantifying HPV16, 18, 31, 33, 35, 39, 45, 51, 52, 56, 58 and 59 [11]. In this application, a cervical or vaginal sample is applied on an indicating FTA elute micro card™ [12–15] and left to dry before storage. The clinical material applied to this card is stable at room temperature and samples stored on FTA cards for 1 year have shown identical HPV typing results as compared to the use of liquid-based cytology (LBC) collection media [16]. Several studies have shown good agreement in HPV testing between samples applied to the FTA card and samples stored in liquid-based media [17, 18]. We have previously demonstrated consistent HPV typing results across menstrual cycle phases and calendar time using the FTA card for sample collection [13]. The HPVIR test is presently used in Uppsala County, Sweden, as triage of women with ASCUS/CIN1 in cytology, and as ‘test of cure’ together with cytology 6 months after excisional treatment of CIN. Since 2008 more than 50,000 samples have been analysed using HPVIR.

One advantage of using the FTA card is their suitability for self-sampling, which represent an important development of cervical cancer screening. Recently, we have shown that repeated self-sampling on FTA cards is as reliable for HPV testing as assisted sampling by a midwife or gynaecologist [14, 15], and that this strategy can result in detection of more than twice as many women with ≥ CIN2 in histology as compared to screening with cytology [14, 15]. In this study we have validated our method of applying samples on the indicating FTA card and using the HPVIR test according to criteria of Meijer and co-workers [2], using collection of LBC samples and the Cobas^®^ HPV test as reference test.

## Methods

### Study population

For this study we invited 896 women who were scheduled for cervical cancer screening in Uppsala County during 2017 and 2018. Among these, 819 women visited a midwife surgery and performed a primary screening test with both methods. Seven hundred ninety-nine women were either HPV negative or had histologically confirmed < CIN2 and were included in the comparison between the candidate test and the reference test to assess the clinical specificity. To determine the sensitivity, we studied a cohort of 75 women scheduled for a colposcopy due to abnormal cytology or/and a positive Cobas® HPV test in primary screening. Of these, 67 women with histologically confirmed ≥ CIN2 were included in the analysis of sensitivity. The inclusion criteria for all participants in the study were ≥ 30 years of age and not hysterectomized, or pregnant.

The included women either had a midwife or a gynaecologist collecting two cervical samples per participant using one cytobrush for each collection. One sample was applied to an indicating FTA elute micro card™ (art. no WB129308, GE Healthcare, Longwood Dr., Cardiff CF14 7YT, UK) and the other sample was transferred to the liquid based medium (LBC) (ThinPrep, Hologic Inc., 250 Campus drive, Marlborough, MA, U.S.A). Sampling was randomized, meaning that the first sample was allocated randomly to either the FTA card or the liquid based medium. The FTA card sample was analysed using the HPVIR test (candidate test) and the LBC sample using Cobas® HPV test (reference test) on the 4800 platform (Roche molecular systems, 4300 Hacienda Dr., Pleasanton, CA 94588, USA). Women with at least one HPV positive outcome of the tests were referred for colposcopy and biopsy. The study was approved by the regional ethics committee in Uppsala, Sweden (Dnr 2017/088).

The inter-laboratory agreement and reproducibility of the candidate test (FTA card and HPVIR test) was determined using samples from 558 women, of which 284 (51%) had previously tested HPV positive using the Hybrid Capture 2 (HC2) test. These samples were originated from another cohort and collected in the Nelson Mandela Hospital (WSU) or Mbekweni Community Clinic, Eastern Cape, South Africa. One sample was collected on the FTA card and the other in a tube of liquid medium for HC2. The laboratory at University of Cape Town (UCT) performed the evaluation of the inter-laboratory agreement. The intra-laboratory reproducibility was studied by comparison of the results of two different research engineers (Operators) in Uppsala, processing the same set of 558 samples.

### HPVIR test of FTA samples

FTA cards were processed using an automated laboratory system (easyPunch STARlet, Hamilton Robotics, Via Crusch 8 CH-7402 Bonaduz, GR, Switzerland) where a robot arm picked up each FTA card, took a photograph of the sample deposition area, and then calculated which part of the card that contained the highest concentration of cells using a machine learning software. The robot first collected 5 punches from a clean card area and thereafter it punched 4 circular pieces of 3 mm diameter from the sample deposit area and collected all 4 punches in a single well in a 96-well microtiter plate. DNA was then extracted as described earlier and the HPV test was performed using the real-time PCR assay HPVIR [11, 19]. The HPVIR test is LDT optimized for the real-time PCR instrument Quantstudio 6 and 7 (Life technologies, 5791 Van Allen Way, Carlsbad, CA 92008, USA) together with specifically tested PCR reagents and oligos. This test detects and quantifies a human single copy gene (housekeeping gene), HMBS (*Homo sapiens* hydroxymethylbilane synthase; GenBank accession no. M95623.1) as a control that the sample contains enough cellular material for the test to be informative. The test also detects and quantifies HPV16, 18, 31, 33, 35, 39, 45, 51, 52, 56, 58 and 59, and provides single genotype information for all types except HPV18/45 and HPV33/52/58, which are detected together as two groups. The limit of detection (LOD) for HPVIR is 10 HPV copies per PCR. In order for a sample to contain sufficient amount of material for the HPV test to be informative, a threshold of 10 copies of the nuclear single copy gene per PCR is used [19].

### Cobas^®^ HPV test on LBC samples

Samples were collected in 20 mL LBC medium and stored in room temperature until analysed. The Cobas^®^ 4800 is a fully automated system for sample preparation and real-time PCR, including the FDA approved Cobas^®^ HPV test. The Cobas^®^ HPV test include 14 hrHPV (16, 18, 31, 33, 35, 39, 45, 51, 52, 56, 58, 59, 66 and 68) of which HPV16 and 18 are detected as single genotypes while the others types are reported as a group denoted ‘Other HPV’ types (12 pooled types). To perform this assay, the 20 mL tube with cervical sample in LBC were loaded into the Cobas^®^ 480 instrument, and 400 μL from each tube was transferred to the extraction plate, after which the samples were lysed in the presence of a chaotropic reagent. The DNA was then purified by adsorption to magnetic glass particles, washed and eluted in 100 μL dH_2_O. Fifty microliter of this was mixed with PCR reagents for amplification in the Cobas^®^ 480 instrument. All Cobas® tests were performed by an accredited laboratory (UniLabs AB, Klinisk molekylärbiologi, Skaraborgs sjukhus, Skövde, Sweden).

### Hybrid capture® 2 assay on liquid-based samples

Samples for the Hybrid Capture® 2 assay (HC2) (Qiagen Str. 1, 40,724 Hilden, Germany) were collected and handled according manufactures recommendations by the lab at University of Cape Town (UCT). HC2 is based on hybridization with RNA probes to detect 13 hrHPV (16, 18, 31, 33, 35, 39, 45, 51, 52, 56, 58, 59 and 68). A cut-off value of RLU/CO = 1, is equivalent to 1 pg HPV DNA per mL sampling buffer was used as a threshold for an HPV positive test.

### LINEAR ARRAY^®^ HPV genotyping

Specimen that were positive with the Cobas® HPV test and typed as ‘Other HPV‘but negative with HPVIR were genotyped using the Roche LINEAR ARRAY^**®**^ HPV genotyping test (Roche molecular systems, 4300 Hacienda Dr., Pleasanton, CA 94588, USA). To this end, 5 mL ThinPrep LBC cervical cell material were centrifuged at 5000 g for 30 min in 4 °C, and the cell pellet was resuspended in 400 μL phosphate-buffered saline. DNA was extracted from resuspended cells using MagNA Pure Compact (Roche) and the MagNA Pure Compact Nucleic Acid Isolation Kit (Roche). HPV genotyping was then performed using the Roche Linear Array HPV genotyping test which identifies 37 different high- and low-risk types (HPV6, 11, 16, 18, 26, 31, 33,35, 39, 40, 42, 44, 45, 51, 52, 53, 54, 56,58, 59, 61, 62, 64, 66, 67, 68, 69, 70, 71, 72, 73, 81, 82, 83, 84, 89 and IS39).

### Colposcopy and pathology

All gynaecologic examinations of Swedish samples were performed at the Clinic of Obstetrics and Gynaecology, Uppsala University Hospital. A sample for cytological analysis was also collected at the same visit. The colposcopic evaluation included an identification of squamocolumnar junction and transformation zone (TZ) with application of 5% acetic acid and iodine solution. Directed biopsies were obtained from all the identified abnormal areas and a blind biopsy was taken in women with normal colposcopy. All cytology and histology samples were analysed at the Clinic of Pathology and Cytology, Uppsala University Hospital, Uppsala. The classification was according to SNOMED (Systematized, Nomenclature of Medicine; College of American Pathologists, Skokie, IL, USA) and the highest histological grade found in each patient was used for interpretation of the results.

### Statistical analysis

Clinical specificity and sensitivity for samples on FTA card analysed with HPVIR was compared with LBC samples analysed with the Cobas® HPV test, using a binomial test for non-inferiority with a power of at least 80%, as estimated in earlier studies [4, 20]. Non-inferiority was defined as a relative sensitivity for ≥ CIN2 of 90%, and a relative specificity for ≥ CIN2 of 98% [4]. In calculating non-inferiority of the specificity, a positive response as described in Tang et al. [20] was defined as a negative test result. Confidence intervals for incidences, sensitivities and specificities were calculated using the modified Wald method as described in Agresti et al. [21]. Kappa values and confidence intervals for kappa values were calculated using the GraphPad online calculator (https://www.graphpad.com/quickcalcs/kappa1/). The thresholds for intra-laboratory reproducibility and inter-laboratory agreement were set to a lower confidence bound of ≥87% with kappa values > 0.5 as proposed by Meijer and colleagues [4].

## Results

### Clinical specificity of the FTA card and HPVIR test

Two samples, the FTA card and LBC, were obtained from 799 women (median age, 42 years, range 30–64 years) with no evidence of ≥ CIN2, and these were used in the calculation of clinical specificity. Of the 799 women, the candidate and reference test agreed on 747 women to be HPV negative and 23 women to be HPV positive, corresponding to an agreement of 96.4% (Table 1). Among the 52 women whom were HPV positive by one or both of the assays, the median follow-up time was 2.0 (range 0.5–4.3) months and 33% (17/52) had normal histology, while 67% (35/52) had CIN1. The incidence of HPV positive women was almost twice as high using the Cobas^®^ HPV test (6.1, 95% CI = 4.6–8.0%, 49/799) compared to using HPVIR (3.3, 95% CI = 2.2–4.7%, 26/799). Among women that were positive only with Cobas® HPV test, 42% (11/26) had normal histology and 58% (15/26) had CIN1. The clinical specificity of HPVIR was 96.8% (95% CI = 95.3–97.8) compared to 93.9% (95% CI = 92.0–95.3) for the Cobas^®^ HPV test. There was no statistical evidence that the clinical specificity of HPVIR was inferior to that of the Cobas^®^ HPV test (T = 5.89, *p* = 1.89 × 10^− 9^).

**Table 1:** Comparison of the HPVIR and COBAS analysis

		Cobas test		
Sample type	HPVIR test	HPV positive	HPV negative	Total
Cases (≥CIN2)	HPV positive	60	4	64
	HPV negative	3	0	3
	Total	63	4	67
Controls (<CIN2)	HPV positive	23	3	26
	HPV negative	26	747	773
	Total	49	750	799

### Clinical sensitivity of the FTA card and HPVIR test

To estimate the clinical sensitivity, samples from 67 women ≥30 years (median age, 37 years, range, 30 to 57 years) with histologically confirmed ≥ CIN2 were used. Among these, 23 women had CIN2 and 44 had ≥ CIN3 and the median follow-up time was 5.2 (range 0 to 42.2) months. Among the 67 women, both tests agreed on 60 women to be HPV positive and none of the women to be HPV negative (Table 1). Four women were HPV positive only with HPVIR and 3 women were HPV positive only with the Cobas^®^ HPV test. No woman was negative by both tests. The agreement between the methods was 89.6% (60/67). The clinical sensitivity of HPVIR was 95.5% (95% CI = 87.2–99.0) and that of Cobas^®^ HPV was 94.0% (95% CI = 85.2–98.1). In conclusion, there was no statistical evidence that the clinical sensitivity of HPVIR was inferior to that of Cobas^®^ HPV test (T = 2.31, *p* = 1.04 × 10^− 2^).

### Analysis of discordant samples

A set (*n* = 23) of the LBC samples that were HPV positive with ‘Other HPV’ using Cobas® HPV test but negative with HPVIR were analysed using the Roche LINEAR ARRAY^**®**^ HPV genotyping test. Of the 23 samples, the linear array was negative for 2 samples, 2 samples had only non-hrHPV types (53, HPV-CP6108, 62), another 2 samples contained both hrHPV and non-hrHPV (6, 54, 61, 62, 83) while the remaining 17 samples had one or several of the following hrHPV 31, 33, 35, 39, 45, 52, 58, 59, 66, 68. Among the 23 samples, 6 contained only HPV66 and/or HPV 68.

### Intra-laboratory reproducibility and inter-laboratory agreement

The intra-laboratory reproducibility and inter-laboratory agreement were estimated using 558 samples, of which 284 were HPV positive by HC2 (51%). In Uppsala, two research engineers (Operators) performed a blinded intra-laboratory test. The reproducibility (kappa value) between operators was estimated to 0.974 (95% CI = 0.956–0.993), with an agreement of 98.7% (Table 2). For the inter-laboratory test samples were collected on the indicating FTA card and analysed by the HPVIR test at the Uppsala and Cape Town laboratories. The inter-laboratory agreement between analyses performed in Uppsala and in Cape Town was 93.7%, with a kappa value of 0.874 (95% CI = 0.834–0.914) (Table 3).

**Table 2:** Intra-laboratory reproducibility of the HVIR test

	Uppsala Operator # 1		
Uppsala Operator #2	HPV positive	HPV negative	Total
HPV positive	235	1	236
HPV negative	6	316	322
Total	241	317	558

**Table 3:** Inter-laboratory agreement of the HPVIR test

	Cape Town Lab		
Uppsala Lab	HPV positive	HPV negative	Total
HPV positive	240	3	243
HPV negative	32	283	315
Total	272	286	558

## Discussion

In this study we compared the clinical performance of cervical samples applied on the FTA card and analysed with HPVIR to LBC samples analysed with the Cobas® HPV test. The clinical sensitivity and specificity of using FTA card and the HPVIR test was not inferior to that of LBC samples and Cobas® HPV test, using the international thresholds of a relative sensitivity = 90% and specificity = 98% [2]. Cervical samples applied to an FTA card and analysed with HPVIR showed intra-laboratory reproducibility close to 1, and a higher inter-laboratory agreement than the recommended 87% [2]. The corresponding kappa values were much higher than 0.5 for both intra- and inter-laboratory comparisons [2].

### Comparisons of HPV test results

Among women without disease, 3.6% (29/799) showed discordant HPV test results between the HPVIR and the Cobas^®^ HPV test, and 89.7% (26/29) of discordant samples were HPV positive only with the Cobas^®^ HPV test. In 88.5% (23/26) of these discordant samples, the genotype was indicated as ‘Other HPV‘by the Cobas^®^ HPV test. For two of these samples, the linear array did not indicate the HPV type, possibly due to limited amounts of material. Of the 21 samples that were Cobas^®^ HPV positive but HPVIR negative and for which the linear array resulted in an HPV genotype, 6 of the samples had HPV66 and 68, types not included in the HPVIR test. HPV66 is a genotype that was previously considered high-risk, but recent studies indicate that it has low or no oncogenic properties [22]. The oncogenic properties of the HPV68 genotype have also been questioned and it has recently been proposed to be an intermediate risk genotype [23]. Three of the samples contained non-hrHPV types to which the Cobas^®^ HPV cross-hybridizes. The category ‘Other HPV‘in the Cobas® HPV test has previously been reported to show cross-reactivity with low-risk HPV genotypes. A study of 5022 women comparing two other commercial hrHPV tests with Cobas® HPV showed that about 25% of the samples that were positive with the Cobas^®^ HPV test represented cross-reactivity with low-risk genotypes [24]. Thus, the category ‘Other HPV‘can include some false positive results. The higher incidence of HPV in women < CIN2 when using Cobas^®^ HPV test compared to HPVIR test would increase colposcopy resources and costs for the screening program, as well as worry some women that will be exposed to unnecessary examination.

The performance of HPVIR and Cobas^®^ HPV was similar in the group of cases used to estimate the sensitivity, were none of the samples was HPV negative and 89.6% (60/67) had HPV positive results. Three samples were positive only using the Cobas^®^ HPV test (3 CIN3, 2 HPV16 and 1 non HPV16/18) and 4 samples (2 CIN2 and 2 CIN3, 1 HPV16 and 3 non HPV16/18) were positive only using the HPVIR test. Our results confirms that HPV16 or 18 occurs in more than 50% of women with high-risk lesions [25].

### Sample collection media

In addition to comparing the HPVIR test against a reference test, we used different sampling media. The internationally widely used standardized method ThinPrep^®^ LBC for the Cobas^®^ HPV test was compared to the indicating FTA Elute™ micro card, the dry sampling medium for the HPVIR test. Since two different samples were collected from each woman, the sampling order may affect the results of the HPV assay. There was no significant difference in HPV positivity between the samples collected first and second for either of the tests. A screening method using a dry storage media as the indicating FTA Elute™ micro card in combination with a highly sensitive HPV test provides an effective method for primary cervical cancer screening. This strategy will present a low cost and high throughput alternative, in addition to being easily adapted for self-sampling. HPV testing using vaginal self-sampling on FTA card has been shown to be as reliable as assisted cervical sampling [14, 15]. We have recently shown that repeating the self-sampling and HPV test for women that were HPV positive in the screening sample after 4–6 months, in order to identify women with persistent infections, can results in more than two-fold higher detection rate of CIN2 + -lesions, compared to cytology screening [14, 15]. This method could decrease both incidence and mortality in cervical cancer, as early detection and treatment remains the most effective means of prevention. By using additional methods of triage of hrHPV positive women, such as methylation status or HPV viral load, the specificity of the screening strategy could be improved, resulting in fewer unnecessary colposcopies and treatments [23].

### Intra-laboratory reproducibility and inter-laboratory agreement

The inter- and inter-laboratory analyses was based on 558 samples collected in South Africa selected on the criterion that at least 30% had previously tested HPV positive with the reference test HC2. In both the intra- and inter-laboratory analyses the lower confidence bounds were > 87% with kappa values > 0.5 and, the results complied well with the guidelines. The HPVIR test has been validated yearly using the WHO panel with 100% proficiency. The result from inter-laboratory testing showed that the HPVIR method is robust enough and can be operated reliably at other sites than Uppsala.

## Conclusions

Cervical samples applied on FTA card and analysed with HPVIR test shows similar sensitivity and specificity as LBC samples analysed with the Cobas^®^ HPV test, and thereby fulfil the requirements of the international guidelines for an HPV test to be used for primary cervical cancer screening. The use of the FTA cards and HPVIR has the advantage that it is both easily used for self-sampling and report extended HPV genotyping results.

## Data Availability

All data generated or analysed during this study are included in this published article.

## Abbreviations

CIN2: Cervical intraepithelial neoplasia grade 2
HC2: Hybrid Capture 2
hrHPV: high-risk human papillomavirus
